# Technical note: Tracking target/chest relationship changes during motion‐synchronized tomotherapy treatments

**DOI:** 10.1002/mp.15667

**Published:** 2022-04-20

**Authors:** William S. Ferris, Wesley S. Culberson, John E. Bayouth

**Affiliations:** ^1^ Department of Medical Physics School of Medicine and Public Health University of Wisconsin‐Madison Madison Wisconsin USA; ^2^ Department of Human Oncology School of Medicine and Public Health University of Wisconsin‐Madison Madison Wisconsin USA

**Keywords:** Radixact, Synchrony, tracking

## Abstract

**Background:**

Radixact Synchrony® is an intrafraction motion tracking system for helical tomotherapy treatments that uses kV radiographs of the target and LEDs on the patient's chest to synchronize the movement of the radiation beam with the respiratory motion of the target. Several works have demonstrated Synchrony's ability to track target motion when the chest and target motions are perfectly correlated.

**Purpose:**

The purpose of this work was to determine Synchrony's ability to accurately adapt to scenarios with a changing target/chest correlation.

**Methods:**

A custom ion chamber mimicking plug with embedded fiducials was placed inside a Delta4 Phantom+ and used as the tracking object. A separate motion stage was programmed to mimic chest motion. The target and chest surrogate phantom were programmed to move sinusoidally and two types of target/chest relationship changes were introduced: rigid shifts and linear drifts of the target position but not surrogate position. Tracking analysis was performed by comparing programmed phantom motion to log files of the Synchrony‐modeled motion. No dosimetry was performed in this work.

**Results:**

At the fastest imaging rate of 2 s/img, Synchrony accurately adapted for gradual drifts in the target location (up to 5 mm/min) with minor increases in tracking errors and adapted for an abrupt 5 mm shift after about 30 s (with an auto‐pause threshold at 60 s). When the imaging period was longer (> 4 s/img), larger tracking errors (> 5 mm) were observed, and the treatment would be paused. The measured delta (MD) parameter (2D target localization error on the most recent image) was found to be a more responsive indicator of tracking errors than the potential difference (PD) parameter (3D estimator of tracking error based on all images in the model). Lastly, the effect of a recent update to the tracking algorithm was found to improve the ability of Synchrony to track target/chest relationship changes.

**Conclusions:**

This work demonstrated that Synchrony can adapt to gradual changes (drifts) in the target/chest relationship, but it takes a finite amount of time to adapt to abrupt shifts. Ability to adapt to these changes increases with increasing imaging frequency. Larger tracking errors were observed in this work than others have reported in the literature due to the introduction of target/chest correlation changes in this work. Future work needs to be performed investigating what type and magnitude of target/chest miscorrelations occur in patients. Lastly, users should ensure they are using the most recent software (3.0.1 or newer) to improve the ability of Synchrony to track these movements.

## 1 INTRODUCTION

Real‐time motion tracking systems are becoming increasingly common in radiotherapy,^1^, ^2^, ^3^, ^4^, ^5^ especially with the implementation of stereotactic body radiation therapy (SBRT), which uses large doses per fraction and thus requires precise target localization.^6^ Radixact Synchrony (Accuray, Inc., Sunnyvale, CA) is a motion tracking system that can be used to correct the delivery for respiratory or nonrespiratory (e.g., quasi‐static motion of the prostate) intrafraction motion.^4^, ^7^ The target is located during the treatment using a kilovoltage (kV) x‐ray tube and detector mounted perpendicular to the megavoltage (MV) source and detector. Images are acquired at 2–6 angles of each gantry rotation. For respiratory motion, light‐emitting diodes (LEDs) are placed on the patient's chest and represent the phase of the patient's respiration.

The ability of Radixact Synchrony to track phantom motion has been demonstrated in the literature.^8^, ^9^, ^10^, ^11^, ^12^ Radixact Synchrony has also been used to treat patients at several institutions.^10^, ^11^ For phantom experiments, Synchrony has been shown to track phantom motion within a root‐mean square (RMS) error of 1.5 mm, and often less than 1.0 mm.^8^, ^9^, ^11^ To be noted is that in this work, “Synchrony” will refer to Radixact Synchrony as opposed to CyberKnife Synchrony.

Respiratory management systems like Synchrony rely on the assumption that the external and internal motions are correlated since they are using movements of the chest as a surrogate for movements of the target. However, Synchrony also addresses correlation changes over time using images acquired throughout the treatment after the initial model is built. There is evidence in the literature that the correlation between the patient's chest and target in radiotherapy may not be strong or may change during a given treatment fraction.^1^, ^13^, ^14^, ^15^, ^16^, ^17^ Malinowski et al. have shown that the relationship between the tumor and the chest surrogate during Synchrony treatments on CyberKnife changed in 22% of lung subjects when observed over 10 min and 67% of subjects when observed over 30 min.^17^

Most of the investigations in the literature on Radixact Synchrony tracking accuracies have been performed with good correlation between the internal (tumor) and external (chest or LED) motion. Ferris et al. investigated tracking errors when there was a phase shift between the target and surrogate motion and found that Synchrony reduces the effects of intrafraction respiratory motion that are not accounted for using the previous motion‐encompassing technique for helical tomotherapy. However, the motions were still perfectly correlated, just shifted in time.^9^ Yang et al. investigated tracking accuracies of Radixact Synchrony and CyberKnife Synchrony for various motions derived from patient treatments, including cases that had a “change in correlation between the target and surrogate motion.”^12^ However, the target location was derived from the chest motion pattern using a CyberKnife Synchrony correlation model,^18^ which inherently makes them correlated. Other experiments have placed the LEDs directly on the phantom, creating a rigid correlation between the LED and target motion.^8^, ^9^, ^10^, ^11^

The purpose of this work was to investigate the ability of Radixact Synchrony to adapt to a changing relationship between the target and surrogate motion. Tracking accuracy measurements were performed using sinusoidal motion patterns with added relationship changes between the target and surrogate sinusoids. Two types of changes were introduced to the target but not surrogate motion: a rigid shift/step and a linear drift. The effect of varying imaging frequencies during treatment and the impact of changing various user‐specified tracking parameters on tracking characteristics was investigated. Lastly, Radixact model version 3.0.1 that includes changes to the tracking algorithm was also investigated to determine the impact of the update with respect to tracking target/chest relationship changes.

## 2 METHODS

### 2.1 The Synchrony respiratory model

There are two types of correlation models used for respiratory motion, namely the linear and elliptical models.^19^ For the linear model, the 3D location of the tumor with respect to the registration position is a function of only the surrogate amplitude. For the elliptical model, the model is also a function of time‐derivative of the surrogate amplitude. The elliptical model intends to account for hysteresis or difference in position at the same tidal volume (or surrogate location) during inhalation and exhalation. The linear and elliptical models require at least 5 and 8 radiographs, respectively, and both models use information from a maximum of 20 radiographs.

Although the precise algorithm is proprietary, the fitted variables are determined from the radiographs in the model, with each radiograph providing 2D information on the location of the target.^19^, ^20^ The fitted parameters are updated after each radiograph. The importance of the information from the radiographs is scaled by the time since that radiograph, such that the oldest radiograph has the least importance in the model. When images are acquired more often, they have more equal weighting since there is less of a difference in image age.

There are several user‐specified parameters that can affect tracking performance: potential difference (PD) threshold, measured delta (MD) threshold, auto‐pause threshold, and fiducial/target detection sensitivity.^19^ PD is an estimator of the 3D tracking error, measured in millimeters. It is a statistical calculation based on all images in the model and the recent LED amplitude data. MD is a measure of how well the model predicted the target location in 2D on the most recent radiograph. It is measured in millimeters at the plane of the target centroid. PD and MD are calculated after each radiograph. The PD and MD thresholds are user‐set values that tell the system to pause if the PD or MD values are larger than the threshold for a certain amount of time. For this work, the thresholds were set to be large (>20 mm) to prohibit frequent pauses, and the PD and MD values were retrospectively analyzed. This allows for determination of the time the treatment would have been paused given smaller thresholds.

The auto‐pause delay is a user‐set grace period that allows treatment to continue and more radiographs to be acquired before pausing the treatment due to inadequate modeling or thresholds being exceeded. The default auto‐pause delay is 10 s, and the maximum is 60 s. If the model pauses from one of these thresholds, the user has the option to reset the model and build an entirely new one or to continue improving the model, which is the default. The effect of changing the auto‐pause threshold on modeling was analyzed. Unless otherwise stated, the auto‐pause delay was set to 60 s to prohibit frequent pauses and allow for retrospective analysis of modeling. The sensitivity of fiducial detection was set to *low* out of the options of low, medium, and high. This value adjusts the confidence threshold above which the fiducial or target detection is determined to be accurately found. Therefore, setting the value to low decreases the likelihood that the fiducials will not be found in an image. Since the highest output kV protocol was used, the contrast of the fiducial projections was high, reducing the probability of a false positive detection. Lastly, the effect of a user‐initiated model reset following a treatment pause was analyzed.

If the confidence in the information obtained from a radiograph is low, that radiograph may be excluded from the model. Low confidence can happen when image quality is low leading to fiducials or the target not being detected, or when the detected target position is far from the expected target position. When a radiograph is excluded from the model, MD is not calculated or displayed to the user and the calculation of PD does not include the information from that radiograph.

Additional properties of modeling have been described by Schnarr et al.^4^ or can be found in the Radixact Physics Essentials Guide (PEG) or Treatment Delivery Manual.^19^, ^20^ Once the model is built, it is then used to compensate the delivery through jaw sway and multi‐leaf collimator (MLC) leaf shifts, which will not be explored in this work.

Following analysis of the initial experiments performed for this work, the vendor implemented our recommended modification to the Synchrony algorithm to be more responsive to changes in the target/chest relationship, including an increased threshold above which images are excluded from the model. The changes will be implemented in Radixact version 3.0.1, which will be released in early 2022. This work analyzed the changes implemented by the vendor by comparing the pre‐3.0.1 model (specifically 3.0.0.10) to the 3.0.1 model. Unless otherwise specified, all data in this work were acquired with the newer 3.0.1 model.

### 2.2 Tracking experiment setup

All measurements in this work were performed using the Synchrony simulation treatment mode which simulates a Synchrony treatment but with no MV beam. Measurements were performed with the fiducial respiratory tracking mode, but the results from this work apply to the fiducial‐free respiratory mode as well provided that the target location is accurately identified on radiographs in both modes. To eliminate the impact of fiducial identification accuracy on modeling, the kV radiograph protocol was set to the highest output setting of 140 kVp and 4 mAs. These conditions represent a clinical scenario in which the probability of target detection is maximized to isolate the ability of the algorithm to adapt to respiratory changes of the patient.

**FIGURE 1:**
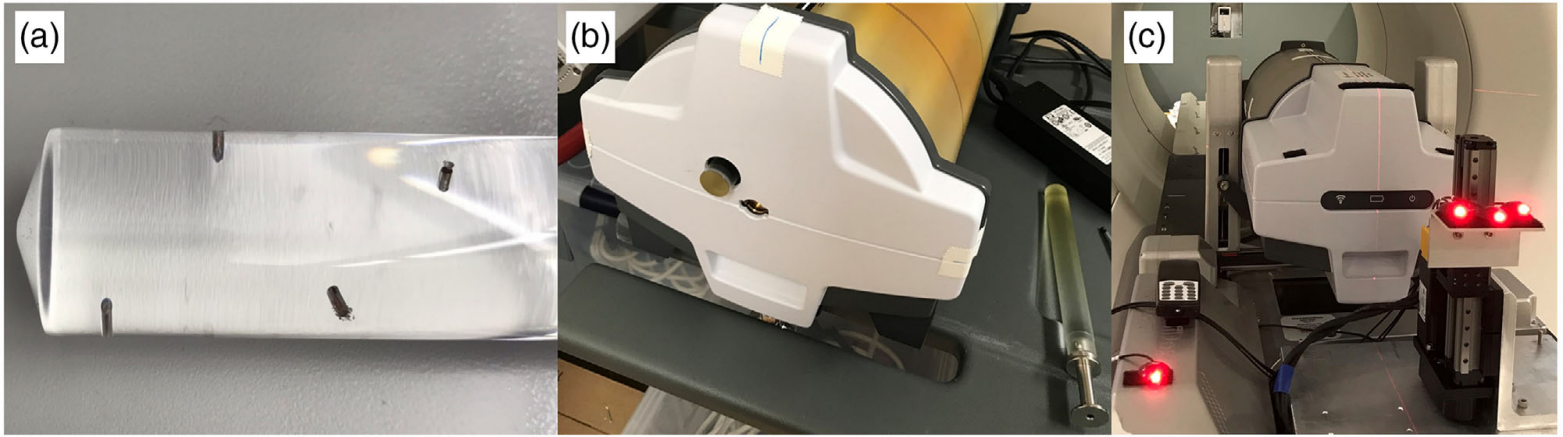
Photographs of (a) the acrylic ion chamber plug insert with embedded gold fiducials used for tracking and (b) the plug inserted into the Delta4 ion chamber slot. The manufacturer's stock ion chamber plug is shown to the right of the phantom. (c) The custom chest‐motion surrogate stage in front of the Delta4

The tracking measurements were performed using a Delta4 Phantom+ (ScandiDos) on a Hexamotion 3D motion stage (ScandiDos, Sweden), shown in Figure 1. A custom acrylic plug with four embedded gold fiducials (3 mm length, 1 mm diameter) was machined to fit into the ion chamber slot of the Delta4. This method of placing tracking fiducials inside the Delta4 allows users with Delta4 phantoms to insert tracking targets into the phantom without making any major modifications. Lastly, a custom surrogate motion stage was designed to hold the LEDs and mimic chest motion. The surrogate motion stage was electrically connected to the drive of the Hexamotion such that the stages could maintain temporal synchronization.

**FIGURE 2:**
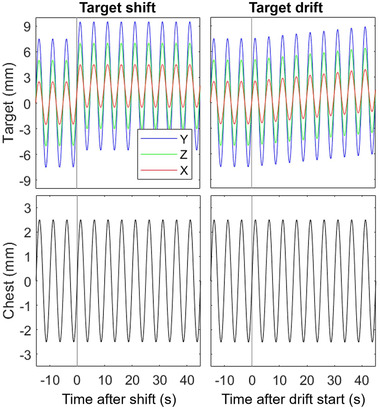
Theoretical cases of adding a 2 mm shift or 2 mm/min drift to the target XYZ motion but not to the chest surrogate

Sinusoidal motion was created with a peak‐to‐peak (PTP) amplitude of 5, 15, and 10 mm for the target in the X, Y, Z directions, respectively, and 5 mm in Z for the surrogate. No phase shifts were present between the motions in each direction. Two types of target/chest relationship changes were investigated in this work: shifts and drifts of the surrogate position. Examples of these changes are shown in Figure 2. Shift or drift magnitudes are specified by the scalar of their 3D vector value, and the component value in each direction is always equal. For example, a 5 mm 3D shift is composed of shifts of ∼2.88 mm in X, Y, and Z. Magnitudes of shifts and drifts ranged from 1.75 mm to 5 mm and 1.75 mm/min to 5 mm/min. In addition, measurements were performed with no shift or drift to be used as a control case for error magnitudes. For all treatments, the model was built and verified to be accurate before the shift or drift started. The accuracy was verified by ensuring on the live visual display that the target motion in each direction agreed with the known PTP amplitudes in each direction and the modeled PD was less than 1 mm.

Measurements were performed using various shift magnitudes, drift speeds, and imaging periods. One measurement setup was repeated three times to assess the repeatability of the results. The imaging period for Synchrony treatments ranges from 2 s/img to 30 s/img and can be varied by changing the number of images per gantry rotation or by changing the gantry rotation period. Note that these are average imaging rates since the images are typically not evenly spaced apart to avoid aliasing.^9^ For this work, the imaging frequency was only varied by changing the gantry period via the pitch to reduce potential variations in tracking that could be introduced from changing imaging angles. Tracking errors were calculated by comparing the known phantom motion to the Synchrony modeled motion, which was obtained from log files after each delivery. The time scales of the curves were aligned by noting phantom trace time at the end of the treatment (therefore the end of the log file), performing approximate alignment by aligning the end point in the log file to the noted phantom trace time, and then fine tuning the alignment by optimizing the time shift to a local minimum in the RMS tracking error. The PD and MD values from each image throughout each treatment were also analyzed, which are the values that are displayed to the user on the console during treatment.

## 3 RESULTS

### 3.1 Effects of imaging frequency and shift/drift magnitude

**FIGURE 3:**
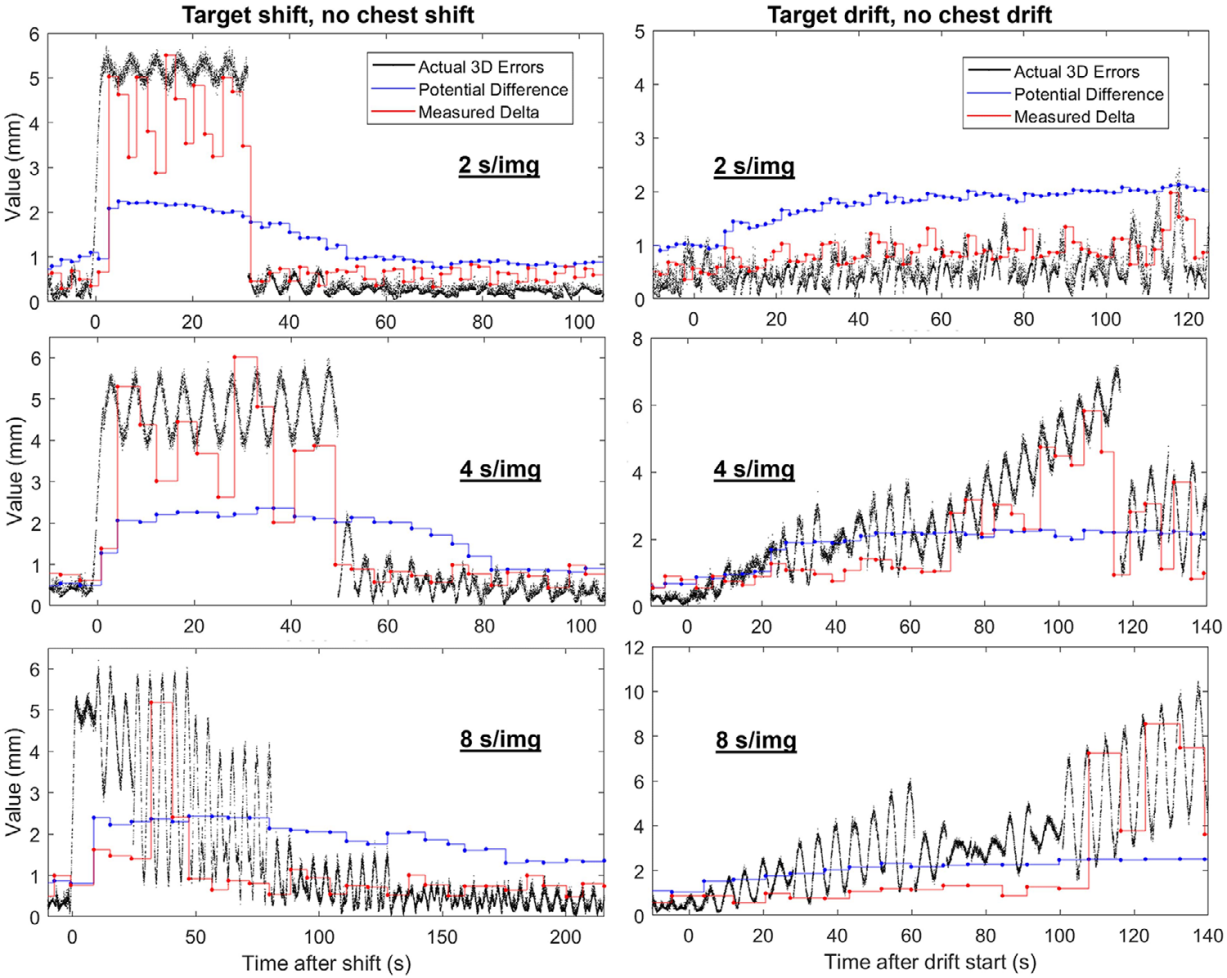
Example tracking after a 5 mm 3D shift (left) or a 5 mm/min 3D drift (right) for various imaging periods. The auto‐pause threshold was 60 s and Radixact version 3.0.1 was used

Table 1 displays results from measurements with varying shift or drift magnitude. Figure 3 displays 3D tracking errors after a 5 mm shift or a 5 mm/min drift in the target position without corresponding shifts or drifts in the surrogate position. The PD and MD values after each radiograph are also displayed on each plot. The maximum tracking error during the control treatment with no shift or drift was 1 mm.

**TABLE 1:** Summary of the results with varying shift magnitude, drift speed, and imaging period. No shifts or drifts in the surrogate were present. Time to correct is the time for the error to return to preshift levels and the maximum error in the 60 or 120 s following the drift start is reported. The auto‐pause delay was 60 s

Set	Imaging period (s/img)	3D target shift magnitude (mm)	Max error (mm)	Time to correct (s)
Control	2	0.00	1.0	–
Shift	2	1.75	2.0	24
	2	3.50	3.8	30
	2	5.00	5.7	32
	4	5.00	6.0	51
	8	5.00	6.2	129

	Imaging period (s/img)	3D target drift speed (mm/min)	Max error after 60 s (mm)	Max error after 120 s (mm)
Drift	2	1.75	1.0	1.0
	2	3.50	1.8	1.8
	2	5.00	1.3	2.4
	4	5.00	3.8	7.2
	8	5.00	6.1	8.5

For the 5 mm shift and imaging period of 2 s/img (the fastest available), the model corrected for the shift after 32 s and errors reached 5.7 mm. For smaller shift magnitudes, the maximum error and the time to correct decreased. For slower imaging periods, the maximum error increased slightly but the time to correct increased largely. For an imaging period of 8 s/img, the magnitude of tracking errors increased from 5.7 mm to 6.2 mm and the time to correct for the shift increased from 32 s to 129 s and the correction was less abrupt. As the imaging period increased, the accuracy of MD in predicting actual 3D errors decreased.

For the 1.75 mm/min drift and 2 s/img imaging period, the maximum error within 2 min after the drift started was 1.0 mm, which is the same as the control with no drift. For the 5 mm/min drift and 2 s/img imaging period, only a slight increase in tracking errors, PD, and MD values were observed relative to before the drift started or the control. The PD was around 1 mm before the drift and plateaued at 2 mm during the drift. The model kept up with the 5 mm/min drift for as long as the measurements were performed, which was about 140 s or 12 mm of total movement. However, when the imaging period was increased, the model was less capable of correcting for the 5 mm/min drift and large tracking errors (> 5 mm) were observed.

### 3.2 Effect of the 3.0.1 update

**FIGURE 4:**
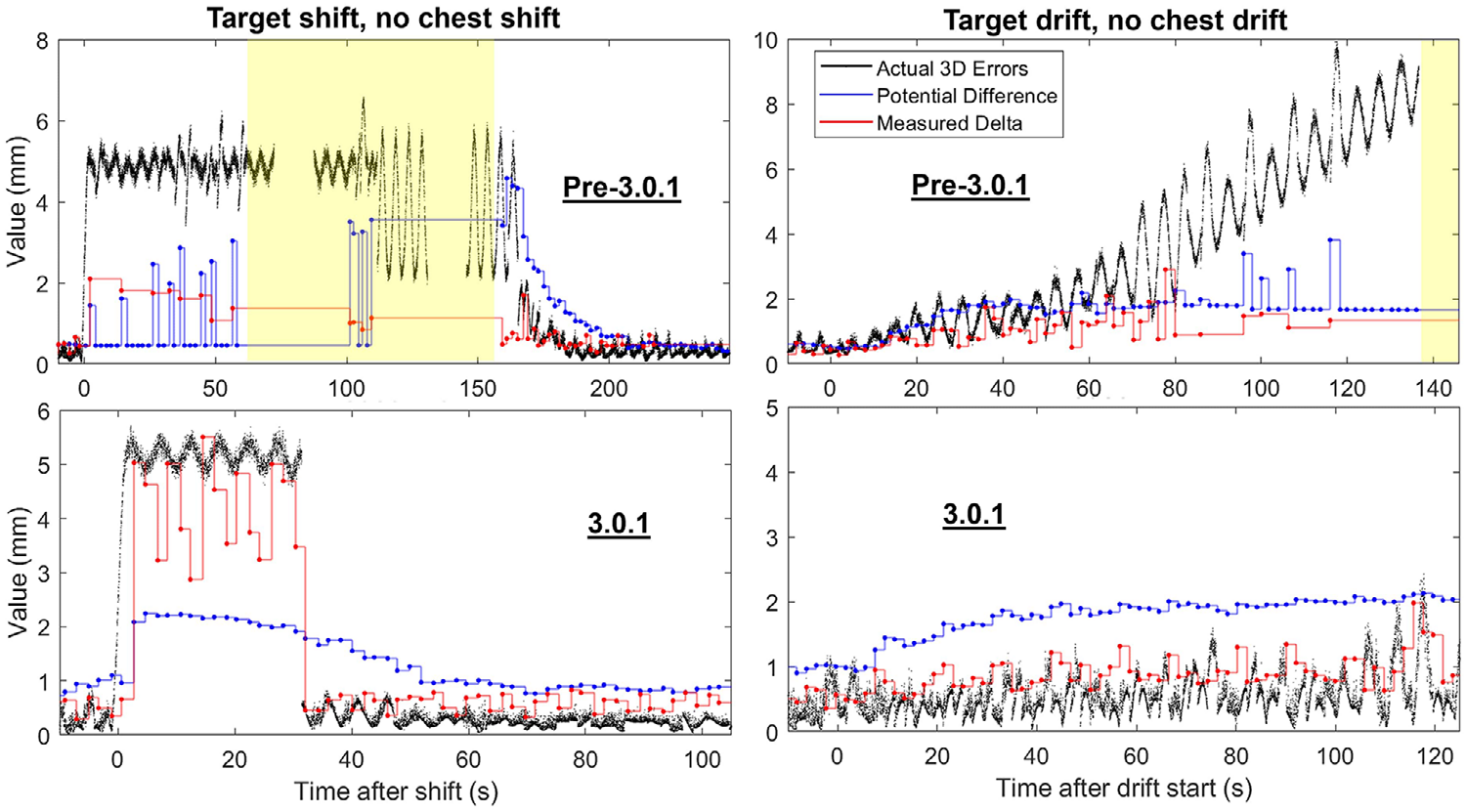
Example tracking after a 5 mm 3D shift (left) or a 5 mm/min 3D drift (right) with an imaging period of 2 s/img for various software versions. The auto‐pause threshold was 60 s. Yellow shading indicates the treatment is paused

Figure 4 displays the results of tracking the same shifts or drifts as in Figure 3 but for the pre‐ and post‐3.0.1 models. The results for 3.0.1 are the same as in Figure 3. The pre‐3.0.1 algorithm took a longer time to correct for the 5 mm shift, required more images to correct, and paused the treatment after 60 s to rebuild the model. The 3.0.1 algorithm corrected for the shift after 40 s with no pauses. Similarly, the pre‐3.0.1 algorithm was not able to keep up with the 5 mm/min drift and eventually paused treatment, but the 3.0.1 model followed the drift with only minor increases in tracking errors.

### 3.3 Effect of auto‐pause threshold and model reset

**FIGURE 5:**
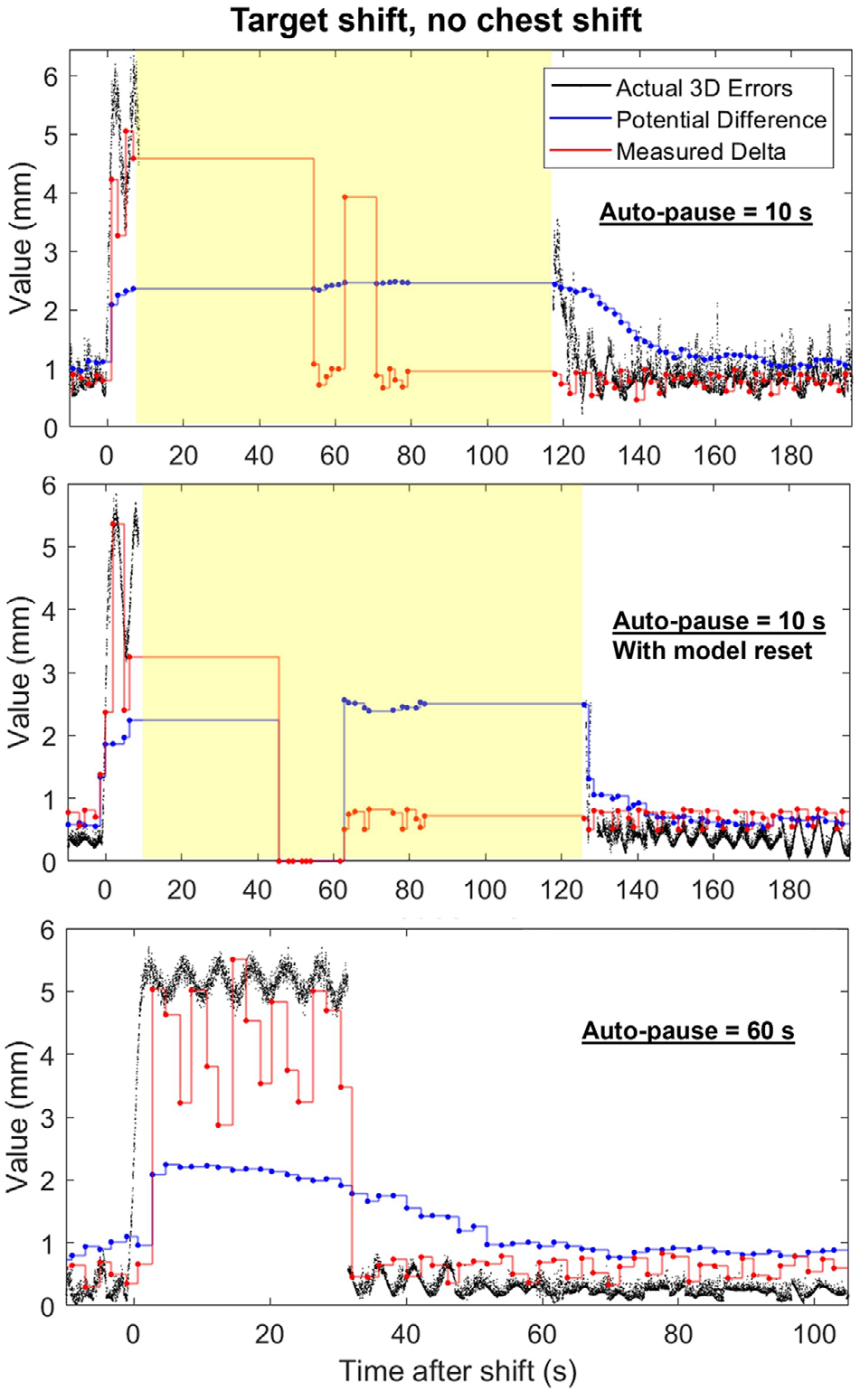
Example tracking after a 5 mm 3D shift with varying auto‐pause thresholds and with a model reset. The imaging period was 2 s/img. Yellow shading indicates the treatment is paused

Figure 5 displays a comparison of tracking a 5 mm shift in the target location with varying auto‐pause thresholds and with or without a model reset following treatment interruption. The results using the 60 s auto‐pause are the same as in Figures 3 and 4. With an auto‐pause of 10 s, the beam turns off 10 s after the shift and allows the user to improve the model with additional images. The system does not store the modeled 3D target location in the log files during the beam‐off and rebuild, which is why no actual 3D error data is displayed during the pause. A total of about 20 radiographs were acquired and 130 seconds transpired (beam on or off) before the correction was made. When a model reset was performed following the treatment pause at 10 s post‐shift, the time to rebuild the model and restart treatment was *longer* than without the reset. These results were confirmed by performing the same experiment multiple times. With an auto‐pause of 60 s, the model corrected after about 40 s (20 radiographs) post shift, and the treatment was active during that time. Therefore, the same number of radiographs were required to correct for the shift in each case. With a shorter auto‐pause, less time is spent with an inaccurate model while the treatment is active, but with a longer auto‐pause, less total time is required to complete the treatment.

## 4 DISCUSSION

To assess the reproducibility of the tracking, the case with the 5 mm 3D shift and 2 s/img imaging period was performed three times. For each trial, the model corrected for the shift after 40±5 s. One of the trials is shown in Figure 3. In addition, the response of the 3D tracking errors, PD, and MD parameters were all visually similar. Variability in tracking results could occur if there were random aliasing between the imaging period and the respiratory period or if the motion of the target or surrogate has random noise. However, the Synchrony algorithm is deterministic, therefore with the same input data the tracking results should be the same. In addition, the repeatability of the mechanical motion of the target and surrogate phantoms is very high since each system has a position tolerance of less than 50 microns. Therefore, the measurements in this work were performed only once other than for this repeatability study.

Synchrony corrected for the 5 mm shift after about 40 s or 20 images. The correction was a discrete change from an inaccurate model (off by 5 mm) to a more accurate model (within 1 mm). The model is built using the most recent 20 radiographs. Once 20 new radiographs were acquired after the shift, the model excluded the preshift radiographs and switched to a new model. The discrete change happens since all the images in the model are more recent (within 40 s), so they all have a higher and more equal weighting. When the imaging period is increased (e.g., 8 s/img), the model takes longer to adapt to the shift, but it adjusts for the shift more gradually. The gradual change is more likely to happen with a larger imaging period because the oldest image (160 s for 8 s/img) has less weighting in the model because of its age.

MD was observed to be a better indicator of tracking performance than PD for the cases in this work. This is because MD is calculated based on a single image and PD is a statistical calculation based on all images in the model. In Figure 3, MD was observed to correlate with actual tracking errors much better than PD, especially when the imaging period is short (≤4 s). When the imaging period is longer, the accuracy of MD in estimating tracking errors decreases. It is uncertain why imaging period affects the accuracy of MD since MD is the raw difference in the predicted vs. actual target position on the most recent radiograph.

Additionally, PD is intended to be an indicator of 3D tracking error, therefore it should agree with the actual 3D tracking errors in Figures 3, 4, 5 better than MD. However, in most cases the magnitude of MD was observed to agree more closely with actual 3D tracking errors than PD. The cases in this work are examples of how actual tracking errors may be much larger than PD, and therefore PD may not always be a conservative estimate of RMS tracking errors as indicated in the literature.^10^, ^19^

This work indicates that when adjusting the user‐set modeling thresholds, there is a trade‐off between time to treat the patient and dose delivery accuracy. Errors during treatment can be reduced by reducing the auto‐pause, PD, and MD thresholds. For example, the amount of time the beam was on during inaccurate modeling in Figure 5 was reduced by reducing the auto‐pause threshold to 10 s. This comes at the expense of a longer total time to complete the treatment. The vendor recommends that the auto‐pause threshold should be set equal to the gantry period such that a full gantry rotation of images will be acquired before the beam is turned off. However, decreasing the thresholds or auto‐pause delay may cause more frequent pauses and increase the total amount of time the patient is in the room, which may not be feasible in a clinical workflow.

The benefit of a model reset following a treatment pause was determined to be small for the investigation in this work. The model reset did not reduce the time required to restart the treatment. This is likely because the minimum time between a treatment pause and acquisition of new images in our experience is 30–40 s, and once a new model is built, it takes another 30–40 s to restart the treatment again. This time reduces the weight of the images before the pauses and puts more weight on the images acquired during the rebuild or after treatment restart, resulting in a decreased weight of the “bad” pre‐pause images, which has a similar effect as a model reset.

The pre‐3.0.1 cases in Figure 4 include examples of Synchrony excluding images from the model. Following the shift, several of the radiographs were excluded from the pre‐3.0.1 model because the modeling confidence in those radiographs was low from the shifted target position. Also, approximately 80 s after the drift started, most of the radiographs were excluded from the model and PD did not accurately increase with increasing tracking errors. Excluded radiographs are indicated on the figure when MD is not displayed, and PD is defaulting to some value and not changing. However, the 3.0.1 model did not exclude any radiographs for the same cases, as observed in Figure 4.

The vendor recommends keeping the gantry period less than 20 s such that the imaging rate would be between 3.33 and 6.67 s/img for 4 to 6 imaging angles. The results from this work agree with this specification. For both shifts and drifts, the Synchrony algorithm (pre‐ or post‐3.0.1) is more capable of correcting for the changes when the imaging period is short. The longer the imaging period, the more time it may take to correct for the target shift and the more dosimetric error may occur. For SBRT treatments which are high‐dose‐per fraction, the 2.5 cm jaw is generally used, and the pitch is very tight (∼0.08), leading to a gantry period of 20–25 s and a resulting imaging period of 4–6 s. It is true that imaging at a short period will increase dose from the kV radiographs. However, previous works have shown that dose from these radiographs is small and the risk of an inaccurate model and misguiding the therapeutic beam are greater than the risks of additional imaging dose.^21^ This work suggests that the imaging period should be considered during the treatment planning process via parameters such as the pitch.

The dosimetric result of poor tracking was not analyzed in this work since the effects of uncompensated motion on helical tomotherapy treatments has been investigated in the literature.^4^, ^7^, ^8^, ^9^, ^22^, ^23^, ^24^ These works indicate that the dose deviations are highly patient specific due to the nature of the interplay effect. For many of the drift cases in this work, the tracking errors were small (< 2.5 mm) or the same as predrift errors, meaning the dosimetric effect will be small. In addition, dose deviations from poor tracking following a shift can be predicted for simple cases. For example, if the target moves 5 mm in the negative *Y* direction (out of the bore), the gantry period is 15 s, and the algorithm takes 30 s to correct for the shift, then axial locations that were in‐field for the previous two gantry rotations may be re‐irradiated and receive a higher dose than planned, and axial locations superior to that which were supposed to be irradiated in the next two gantry rotations may receive a lower dose than planned. It is expected that the magnitude of the tracking errors will impact the magnitude of dosimetric errors. Tighter tracking parameters (PD threshold, MD threshold, auto‐pause) can be used to decrease dosimetric errors.

There is evidence in the literature that the target/chest correlation is not perfect.^1^, ^13^, ^14^, ^15^, ^16^, ^17^ However, there are limitations with these studies. First, they are limited by sample size. In addition, some only report correlation coefficients between the target and chest position over time, which ignores temporal information. Lastly, some studies obtain target information that is derived from chest motion and a correlation model, which inherently creates a correlation between the two.^1^, ^18^ Additional data quantifying the magnitude of the changes could help inform what target margins should be added to account for these target/chest miscorrelations. This requires real‐time, high‐frequency imaging of the tumor and chest over several minutes for a large cohort of subjects, which is difficult to obtain. Overall, the current work shows that the specification that Synchrony always locates the target within 1.5 mm^8^, ^9^ may not be a good assumption once target/chest relationship changes are considered.

This works suggests that the LEDs should be placed on a separately programmable surrogate stage that mimics chest motion when testing the capabilities of tracking systems such as Synchrony. There are a few problems with placing the LEDs on the target phantom or on a perfectly correlated platform. First, the amplitude of LED motion can be unrealistically large when they are placed on the target phantom itself. Second, when the LEDs are on the target phantom, the study only tests the ability of synchrony *to build* a model, not to adapt the model when the correlation between the chest and target changes other than changes in the direction of the target motion. However, placing the LEDs on the phantom still tests the dosimetric components of Synchrony including the jaw sway and MLC shifts.

## 5 CONCLUSIONS

In this work, the ability of the Synchrony system to adapt to a changing target/chest relationship was explored using sinusoidal phantom motion. The system was able to keep up with gradual drifts in the target/chest relationship with minor increases in tracking errors when imaging at the fastest imaging frequency. However, it required about 20 images acquired over 40 s to correct for a 5 mm abrupt shift of the target position with no shift in the chest surrogate position. The ability to compensate for these changes increases with increasing imaging frequency, which indicates that short gantry periods should be used for these treatments since images are acquired at a set number of angles each gantry rotation. Higher sensitivity to pauses from user‐set thresholds increases patient safety but also increases the frequency of treatment interruptions and the total time required to treat the patient. A recent update to the tracking algorithm was found to increase the system's responsivity to changes in the target/chest correlation. Future work must be performed to investigate what target/chest relationship changes are occurring in patients, as the current data available are limited.

## CONFLICT OF INTEREST

John E. Bayouth has ownership interest in MR Guidance, LLC, which has business activity with a company that utilizes image guided radiation therapy technology (ViewRay, Inc.). While this project was not sponsored externally, the data was collected on a Radixact system (Accuray, Inc.) provided to UW‐Madison under a research agreement (Bayouth, PI).

The remaining authors have no conflicts of interest to disclose.
